# Human Anthrax Transmission at the Urban–Rural Interface, Georgia

**DOI:** 10.4269/ajtmh.15-0242

**Published:** 2015-12-09

**Authors:** Ian Kracalik, Lile Malania, Paata Imnadze, Jason K. Blackburn

**Affiliations:** University of Florida, Gainesville, Florida; National Center for Disease Control and Public Health, Tbilisi, Georgia

## Abstract

Human anthrax has increased dramatically in Georgia and was recently linked to the sale of meat in an urban market. We assessed epidemiological trends and risk factors for human anthrax at the urban–rural interface. We reviewed epidemiologic records (2000–2012) that included the place of residence (classified as urban, peri-urban, or rural), age, gender, and self-reported source of infection (handling or processing animal by-products and slaughtering or butchering livestock). To estimate risk, we used a negative binomial regression. The average incidence per 1 million population in peri-urban areas (24.5 cases) was > 2-fold higher compared with rural areas and > 3-fold higher compared with urban area. Risk from handling or purchasing meat was nearly 2-fold higher in urban areas and > 4-fold higher in peri-urban areas compared with rural area. Our findings suggest a high risk of anthrax in urban and peri-urban areas likely as a result of spillover from contaminated meat and animal by-products. Consumers should be warned to purchase meat only from licensed merchants.

Anthrax, caused by the bacterium *Bacillus anthracis*, is a widely distributed zoonotic disease that primarily afflicts herbivorous animals.^1^ Human transmission is typically associated with rural agricultural activities such as slaughtering cattle or industrial processing.^1^ However, anthrax outbreaks and the spread of infection have also been documented in urban markets and livestock trading centers from the illegal sale of contaminated animal by-products.^2^,^3^

In Georgia, the incidence of anthrax has increased dramatically (> 5-fold during 2010–2012) and expanded geographically; evidence suggests urban areas were also at high risk.^4^,^5^ Recently, human anthrax was linked to the sale of contaminated meat at an urban market in Tbilisi,^6^ the Georgian capital, highlighting the potential for disease spillover into uncharacteristic areas at risk for anthrax transmission. In this instance, the sale of meat occurred at the Navtlugi market in the Isani District without undergoing proper inspection; it was then transported ~12 km to the Dezertirebi agrarian market in Tbilisi, where the meat was resold.^6^ An individual subsequently contracted cutaneous anthrax after preparing the purchased meat for consumption; an epidemiological investigation traced the meat back to the informal meat merchant and halted sales.

Given this recent event and the status of the disease in the country, we assessed epidemiological characteristics of human anthrax at urban–rural interface during the period 2000–2012 in Georgia.

We reviewed epidemiologic records from the National Centers for Disease Control and Public Health that included the case patients' place of residence, age, gender, and self-reported source of infection. Place of residence was mapped at the village level and classified as either urban (> 800 people/km^2^), peri-urban (800–250 people/km^2^), or rural (< 250 people/km^2^) using population estimates from the World Population Mapping Project (WorldPop; http://www.worldpop.org.uk/) in ArcGIS (Esri, Redlands, CA) (Figure 1A

**Figure 1.**
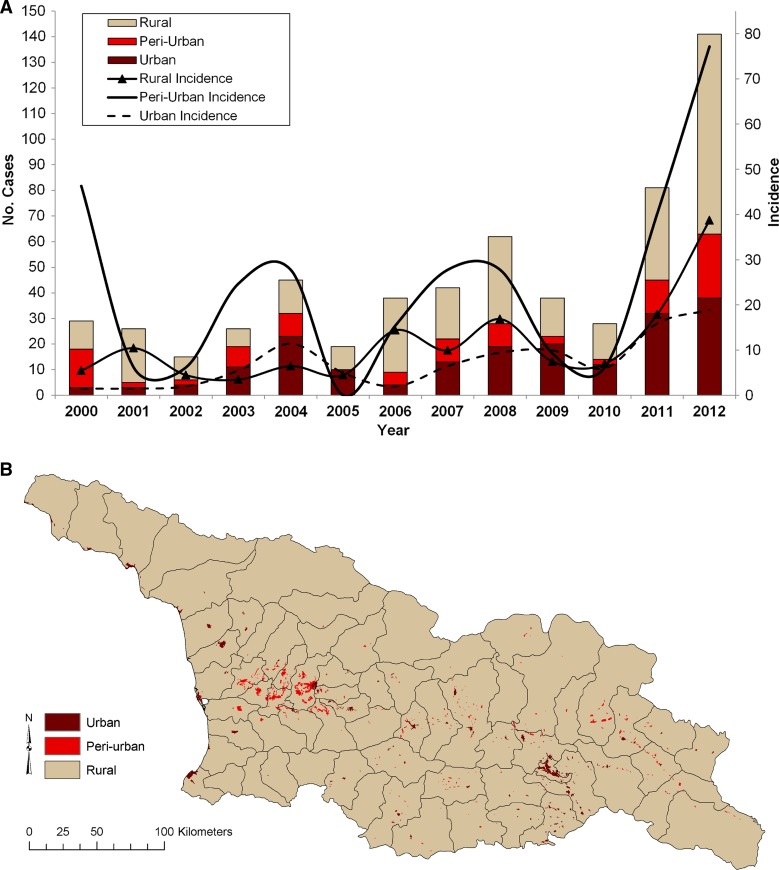
(**A**) Annual trends in the occurrence of rural, peri-urban, and urban human cutaneous anthrax cases in the Republic of Georgia, 2000–2012, (**B**) geographic distribution of urban, peri-urban, and rural areas in Georgia using population estimates from the World Population Mapping Project (WorldPop; http://www.worldpop.org.uk/).

). Annual incidence rates per 1 million person-years were calculated for urban, peri-urban, and rural areas using Georgian national census data (GeoStat, www.geostat.ge) and WorldPop estimates.

Associations between the classified place of residence and self-reported source of infection were analyzed using a χ^2^ test in SAS (SAS Institute, Cary, NC; PROC FREQ). We estimated the risk associated with urban, rural, and peri-urban communities and assessed two self-reported sources of infection: slaughtered/butchered livestock and handled/processed/purchased meat or livestock by-products. We used a generalized linear model (GLM) with a negative binomial distribution in SAS (PROC GLM); because of overdispersion in the number of anthrax cases (ratio of the mean/variance was > 1) a negative binomial distribution was selected over a Poisson distribution.^7^ We ran two models: model 1 with case patients' risk factors associated with slaughtering/butchering and model 2 with risk factors associated with handling/processing/purchasing meat. Risk factors included age, gender, and community classification (urban, peri-urban, or rural). Incidence risk ratios (IRRs) were derived for each variable by exponentiation of the GLM model coefficients (SAS Institute; PROC GENMOD). We ran two separate regression models since risk varied across levels of the classified place of residence and the self-reported source of infection.

During 2000–2012, there were 592 reported cases of human cutaneous anthrax in Georgia (Figure 1); of these, 299 (51%) were classified as rural, 103 (17%) were peri-urban, and 190 (32%) were urban. Case reporting fluctuated between years with high peri-urban reporting in 2000; high urban reporting in 2003, 2004, and 2009; and predominantly rural reporting in the remainder of the time. For the 13-year period, the average incidence/million (95% confidence interval [CI]) in peri-urban areas (24.5 cases/peri-urban population, 95% CI: 13.5, 35.4) was > 2-fold compared with that in rural areas (11.4 cases/rural population, 95% CI: 6.3, 16.5), and > 3-fold compared with that in urban areas (7.3 cases/urban population, 95% CI: 4.4, 10.1).

Of the 592 cases, 497 (84%) reported either an exposure from slaughtering/butchering livestock (318 cases) or handling/processing meat or animal by-products (179 cases) (Table 1). Of the cases that reported exposure from handling/processing/purchasing meat, 100 (56%) reported purchasing meat. The proportion of self-reported exposures differed between rural, peri-urban, and urban areas (χ^2^ = 49.3, df = 2, *P* < 0.001); slaughtering/butchering livestock was more common in rural areas (78% [174]) and peri-urban areas (67% [70]) compared with urban areas (44% [74]).

IRR estimates are shown in Table 2. In model 1 (butchering/slaughtering), rural areas were associated with higher risk compared with urban areas (IRR: 0.44, 95% CI: 0.34, 0.58) and at lower risk compared with peri-urban areas (IRR: 2.36, 95% CI; 1.79, 3.13), adjusting for other factors. In model 2 (handling/processing), rural areas were associated with lower risk compared with urban areas (IRR: 1.91, 95% CI: 1.35, 2.70) and peri-urban areas (IRR: 4.27, 95% CI: 2.77, 6.59), adjusting for other factors.

We provide preliminary evidence of epidemiologic differences in human anthrax risk related to the place of residence in Georgia. Our findings indicated that reported exposure risks varied among rural, peri-urban, and urban areas. Transmission of human anthrax is typically associated with rural agriculture and slaughtering of livestock, as documented in Turkey.^2^,^8^,^9^ In contrast, the spread of cases have also been linked to the sharing or selling of meat; in Paraguay, > 90% of cases were linked to the carrying of meat among individuals not involved with slaughtering or butchering. Consistent with these findings, we documented a majority of cases that reported slaughtering or butchering livestock; however, we showed a higher risk from handling/processing/purchasing meat or animal by-products in urban and peri-urban areas compared with rural areas in Georgia. One hypothesis to explain the high urban risk in Georgia is the spillover of anthrax across the urban–rural interface from the sale or sharing of contaminated meat and animal by-products; the recent dramatic increase in anthrax in Georgia has likely facilitated this process.^4^

Although reports of urban anthrax are uncommon, human transmission has been documented in urban areas of Brisbane (Australia),^10^ Almaty (Kazakhstan)^6^,^11^ and in Europe from injection drug use.^12^ Informal or illegal meat markets are often used to sell contaminated livestock by-products or meat to recoup economic losses.^3^ Agrarian markets and livestock production are often situated at the fringes of urban areas where they are more accessible, possibly explaining the high peri-urban incidence we observed. In Ukraine, informal meat markets are a common occurrence, including major cities such as Kyiv (M. Bezymennyi, personal communication). Anthrax was recently confirmed in Ukraine in a backyard dog that was fed contaminated meat,^13^ and that same contaminated meat was illegally sold at an urban market.^14^ As were previously documented, urban outbreaks in Tbilisi in 1995 and again in 1999 likely involved the sale and distribution of contaminated meat; the latter outbreak involved up to 42 individuals.^15^ Our findings substantiate an earlier study that suggested contaminated meat sales were associated with the geographic clustering of human anthrax around urban areas in Georgia^4^ and are also in keeping with research linking the spread of human anthrax between communities and transnationally via the sharing or sale of infected meat.^5^,^16^

Changes to veterinary health policy and the cessation of compulsory livestock vaccination in the mid-2000s have also likely contributed to the current situation. Efforts to increase the number of official slaughtering plants may help ease barriers to slaughterhouse access and reduce the occurrence of illegal “shade tree” livestock slaughtering. However, indemnity programs that reimburse all or part of a sick or dying animal's value may go a long way in alleviating the economic burden.

The true level of exposure risk in urban areas is unknown since handling and cooking *B. anthracis*-contaminated meat may not lead to clinical infection.^17^ Classifying urban and rural communities is difficult. Although we used established methodologies from the scientific literature,^18^ our technique may have misclassified some communities. Additional research is needed to corroborate epidemiological records with geographic patterns of transmission.

More stringent regulations and education about the disease are needed as agricultural retail products that bypass inspection and purchasing meat via informal markets without knowledge on the condition of the animal may increase risk.^16^ Sustained livestock vaccination campaigns remain the most effective way to reduce human anthrax as shown elsewhere in the region,^19^ and efforts may be needed in or around uncharacteristic hot spots such as urban areas. Consumers should be warned to purchase meat only from licensed merchants with proper documentation.

## Figures and Tables

**Table 1:** Demographic characteristics of human anthrax cases in Georgia, 2000–2012

Classification*	Age†	Sex	Self-reported source of infection	
			Slaughter/butcher	Handle/process/cook
Rural	44 (42, 46)	M	152	19
		F	22	30
Peri-urban	42 (40, 45)	M	63	16
		F	7	19
Urban	47 (45, 49)	M	70	46
		F	4	49

* Place of residence was classified as either urban (> 800 people/km^2^), peri-urban (800–250 people/km^2^), or rural (< 250 people/km^2^) using population estimates from the World Population Mapping Project (WorldPop; http://www.worldpop.org.uk/).

† Mean age (95% confidence intervals).

**Table 2:** Results of the negative binomial regression models examining case patient risk factors for human cutaneous anthrax in Georgia, 2000–2012

Characteristic	Univariate IRR*	Adjusted IRR*	95% CI†	*P* value
Model 1: slaughtering/butchering livestock				
Age (years)				
5–19	0.08	0.09	0.05, 0.15	< 0.001
20–34	0.72	0.57	0.42, 0.78	< 0.001
35–49	0.91	0.85	0.64, 1.14	0.28
50–64	1 (ref.)	1 (ref.)	–	–
Gender				
Female	1 (ref.)	1 (ref.)	–	–
Male	14.32	11.02	7.60, 16.13	< 0.01
Community classification				
Rural	1 (ref.)	1 (ref.)	–	–
Peri-urban	2.52	2.36	1.79, 3.13	< 0.001
Urban	0.39	0.44	0.34, 0.58	< 0.001
Model 2: handling/purchasing meat and animal by-products				
Age (years)				
5–19	0.06	0.08	0.04, 0.15	< 0.001
20–34	0.39	0.29	0.19, 0.45	0.03
35–49	0.60	0.62	0.43, 0.87	< 0.01
50–64	1 (ref.)	1 (ref.)	–	–
Gender				
Female	1 (ref.)	1 (ref.)	–	–
Male	0.96	0.98	0.73, 1.32	0.89
Community classification				
Rural	1 (ref.)	1 (ref.)	–	–
Peri-urban	4.98	4.27	2.77, 6.59	< 0.001
Urban	2.26	1.91	1.35, 2.70	< 0.001

CI = confidence interval; IRR = incidence risk ratio; ref. = referent.

* χ^2^ goodness-of-fit test indicated that the models fit the data (df = 31, χ^2^ = 40.71, *P* = 0.11; df = 31, χ^2^ = 38.28, *P* = 0.15).

† Wald 95% CIs.
